# Editorial: Translational research of occupational therapy and neurorehabilitation, volume II

**DOI:** 10.3389/fnhum.2024.1426481

**Published:** 2024-05-29

**Authors:** Ryouhei Ishii, Hikari Kirimoto, Masafumi Yoshimura, Takayuki Tabira

**Affiliations:** ^1^Department of Occupational Therapy, Osaka Metropolitan University Graduate School of Rehabilitation Science, Habikino, Japan; ^2^Department of Psychiatry, Osaka University Graduate School of Medicine, Suita, Japan; ^3^Department of Sensorimotor Neuroscience, Graduate School of Biomedical and Health Sciences, Hiroshima University, Hiroshima, Japan; ^4^Department of Occupational Therapy, Faculty of Rehabilitation Kansai Medical University, Hirakata, Japan; ^5^Graduate School of Health Sciences, Kagoshima University, Kagoshima, Japan

**Keywords:** occupational therapy, neurorehabilitation, movement-related cortical potentials (MRCP), biofeedback, high-definition transcranial direct current stimulation (HD-tDCS), transcutaneous auricular vagus nerve stimulation (taVNS), phantom limb pain

Occupational therapy (OT) plays a pivotal role in neurorehabilitation, aiming to enhance individuals' ability to engage in meaningful activities and daily life tasks. As advancements in neuroscience continue to unravel the complexities of brain function and rehabilitation, it is imperative for OT practitioners to stay abreast of emerging evidence and innovative interventions. In this editorial, we summarize and discuss recent research findings in the field of neurorehabilitation, with a focus on the clinical implications from an occupational therapy perspective.

The study by Ogahara et al. underscores the importance of ecological validity in assessing cortical activity associated with motor skill learning. By comparing movement-related cortical potentials (MRCP) between real and simulated movement tasks, the researchers shed light on the differences in cortical activation patterns. Occupational therapists can glean insights from this study to design interventions that closely mimic real-life scenarios, thus enhancing the transfer of motor learning to functional tasks.

The next two articles address the application of new technologies to stroke. Marin-Pardo et al.'s investigation into the use of muscle biofeedback in chronic stroke survivors highlights the potential of at-home telerehabilitation for individuals with severe upper limb impairment. Their study demonstrates promising results in improving motor control and functional outcomes, highlighting the potential of telerehabilitation as a more accessible and effective option for individuals with limited movement. The findings underscore the importance of accessibility and engagement in rehabilitation, offering a promising avenue for OT practitioners to leverage technology to deliver effective interventions tailored to individual needs.

Williamson et al. studied on high-definition transcranial direct current stimulation (HD-tDCS) for upper extremity rehabilitation post-stroke presents a novel approach to modulating cortical excitability. Their findings suggest that HD-tDCS can improve motor function by facilitating the lesioned corticospinal tract and inhibiting hyperexcitability in the contralesional cortico-reticulospinal tract. OT practitioners can explore the integration of non-invasive brain stimulation techniques into neurorehabilitation protocols, complementing traditional therapeutic approaches to optimize outcomes for individuals with moderate-to-severe motor impairments.

Aljuhani et al.'s examination of transcutaneous auricular vagus nerve stimulation (taVNS) paired with oral feeding in infants provides valuable insights into early neurodevelopmental outcomes. Their study highlights the potential of taVNS to improve sensory processing in infants with feeding difficulties, offering insights into novel interventions for neurodevelopmental disorders. Occupational therapists can consider the potential impact of sensory processing on feeding behaviors and developmental trajectories, informing holistic intervention approaches for infants at risk of feeding difficulties.

Yoshimura et al.'s case report on virtual reality training for phantom limb pain underscores the importance of addressing sensory-motor integration in chronic pain management. Their findings suggest that VRT can reduce pain intensity and increase upper limb activity on the amputated side, offering a promising approach for managing PLP. Occupational therapists can explore immersive technologies to provide multisensory stimulation and facilitate sensorimotor reintegration, thereby alleviating pain and improving functional outcomes in individuals with limb loss.

Tazaki's review on neurofeedback training for patients with mild cognitive impairment (MCI) and Alzheimer's disease (AD) highlights the potential of non-invasive interventions to enhance cognitive abilities. Occupational therapists can collaborate with interdisciplinary teams to incorporate neurofeedback techniques into comprehensive rehabilitation programs, catering to the cognitive needs of aging populations.

Occupational therapists play a vital role in translating research findings into practical interventions that promote functional independence and quality of life for individuals with neurological conditions. These studies offer valuable insights into novel interventions and assessments in neurorehabilitation, emphasizing the importance of evidence-based practice and interdisciplinary collaboration in optimizing outcomes for individuals with neurological conditions. Occupational therapists are well-positioned to integrate these advancements into their practice, providing holistic and client-centered care to promote meaningful participation and quality of life. By staying informed about emerging research and embracing new approaches, occupational therapists can continue to advance the field of neurorehabilitation and enhance outcomes for their clients.

## Author contributions

RI: Supervision, Validation, Visualization, Writing – original draft, Writing – review & editing. HK: Methodology, Project administration, Resources, Writing – original draft. MY: Data curation, Formal analysis, Funding acquisition, Writing – review & editing. TT: Data curation, Formal analysis, Funding acquisition, Writing – original draft, Writing – review & editing.

